# Social Anxiety and Suicidality in Youth: A Systematic Review and Meta-analysis

**DOI:** 10.1007/s10802-022-00996-0

**Published:** 2022-12-16

**Authors:** Eleanor Leigh, Kenny Chiu, Elizabeth D. Ballard

**Affiliations:** 1 Department of Experimental Psychology, University of Oxford, Oxford, UK; 2 Department of Clinical Psychology and Psychological Therapies, University of East Anglia, Norwich, Norfolk, UK; 3 Experimental Therapeutics and Pathophysiology Branch, National Institute of Mental Health, National Institutes of Health, Bethesda, MD USA

**Keywords:** Suicide, Social anxiety, Social phobia, Adolescent, Youth, Meta-analysis

## Abstract

Suicide is a leading cause of death among young people every year. Identifying risk factors provides opportunities to intervene, and social anxiety may represent one such factor. This systematic review and meta-analysis aimed to review the evidence of associations between social anxiety and suicidality in youth (10–25 years). Embase, PsycInfo, and Medline were searched to identify relevant articles. Meta-analysis was conducted to examine the mean effect sizes of concurrent and prospective associations between social anxiety and three indices of suicidality in adolescents aged 10–25 years. Meta-analyses of 16 studies showed that social anxiety was associated cross-sectionally with suicide attempt (*r* = 0.10, 95% CI: 0.04, 0.15), suicidal ideation (*r* = 0.22, 95% CI: 0.02, 0.41), and suicide risk (*r* = 0.24, 95% CI: 0.05, 0.41), and prospectively at trend level with suicidal ideation (*r* = 0.62, 95% CI: -0.03, 0.90). An examination of the prospective associations with suicide attempt and risk was not possible due to a lack of studies. Several studies suggested that results could not be solely attributed to depressive symptoms. A high level of heterogeneity was observed in each meta-analysis. Moderation analysis was possible for gender and publication year only; neither was significant. Findings provide further evidence of a link between social anxiety and suicidal thoughts and behaviors in youth but are limited by the small number of studies of mixed quality. This review supports future research into social anxiety symptoms as potential risk factors and treatment targets for suicidal youth.

**Supplementary Information:**

The online version contains supplementary material available at 10.1007/s10802-022-00996-0.

## Introduction

Almost 800,000 people die by suicide every year, and it is the fourth leading cause of death among young people globally (World Health Organization, 2019). Suicide attempts are more common than death by suicide, with approximately 2% of the population attempting suicide at some point in their life (Turecki & Brent, 2016). Identifying risk factors for suicidal behaviors in youth (aged 10–25 years) may provide important opportunities to intervene.

Psychiatric disorders are a well-known risk factor for suicidal behavior. Retrospective studies in which family members have been interviewed have reported the presence of a psychiatric disorder in 90% of individuals who die by suicide (Arsenault-Lapierre et al., 2004). Disorders such as major depressive disorder, bipolar disorder, and personality disorders have been shown to have the strongest association with suicidal behavior (Turecki & Brent, 2016). However, anxiety disorders are also associated with increased risk, particularly in the transition from suicidal thoughts to suicide attempt (Nock et al., 2010). Epidemiological adult samples have demonstrated that each anxiety disorder is associated with increased risk of lifetime suicidal thoughts and behaviors, independent of other mood or substance use disorders (Thibodeau et al., 2013). A review and meta-analysis by Bentley et al. (2016) examined 65 studies of adults and adolescents and reported significant albeit weak associations between anxiety disorders and suicidal ideation (OR = 1.49, 95% CI: 1.18, 1.88) and suicidal attempt (OR = 1.64, 95% CI: 1.47, 1.83).

Social anxiety disorder (SAD) might be particularly relevant for suicide risk due to its overlap with key suicide risk factors. SAD is a common mental health condition, affecting about 11% of the population over the lifespan and typically starting in adolescence (Kessler et al., 2005). It is characterised by a marked and persistent fear of being humiliated or rejected by others, and often linked to feelings of social isolation and loneliness (Alden & Taylor, 2004). According to the interpersonal theory of suicide (Joiner, 2007), perceived burdensomeness and thwarted belonginess confer vulnerability for suicidality (e.g., Joiner et al., 2009; Van Orden et al., 2008), which are also characteristics of SAD. Indeed, SAD has been identified as a key diagnosis in the familial aggregation of suicidal behavior, even after adjusting for other mood disorders (Ballard et al., 2019). In line with this, the review of Bentley et al. (2016) identified SAD as a risk factor for both suicidal ideation and attempt. However, only one of the 11 studies in the review focused on adolescents (Gallagher et al., 2014) and more studies have been published since then. A review of studies examining the association between SAD and suicidal thoughts and behaviours in adolescents specifically is warranted because SAD represents the peak period of SAD onset (Kessler et al., 2005). This would have important clinical implications in view of the possibility that better early treatment of the condition could reduce the risk of later suicidal behavior.

Social anxiety is highly comorbid with depression (Wittchen et al., 1999) with more than 20% of those with social anxiety disorder suffering from depression at the same time (Dunner, 2001). It is possible that an observed association between social anxiety and suicidality is an artefact of depression. We aimed to test this possibility in our review. We also aimed to examine possible moderators including clinical and demographic factors. The study aims were to examine in adolescents aged 10–25 years: (1) the concurrent association between social anxiety and its symptomatology, with lifetime suicide attempt, current suicidal ideation, and current suicidal risk; (2) the prospective relationship between social anxiety with suicide attempt, suicidal ideation, and suicidal risk; (3) the specificity of these associations, over and above depression symptoms; and (4) moderators of these associations, such as clinical and demographic factors. Age will be one demographic factor examined due to the important potential developmental differences in the link between social anxiety and suicidality. For the purposes of this review, suicidal ideation is defined as active or passive thoughts about dead, or wanting to be dead, with any method, plan or intent. Suicidal behavior is defined as a potentially self-injurious behavior associated with at least some intent to die. Suicide risk refers to the likelihood of an individual to attempt or die by suicide. Likelihood is estimated by assessing the presence of predisposing and precipitating factors and their interactions (O'Connor & Nock, 2014; Van Orden et al., 2010). We used the word “current” to describe variables measured at baseline.

## Methods

### PROSPERO Registration

The systematic review was pre-registered on PROSPERO (reference: CRD42021248538) prior to literature search.

### Search Strategy

The review conforms to the PRISMA statement (see Supplementary Materials, Appendix 1 for the PRISMA checklist). Studies published from inception to 11 February 2022 were retrieved from Embase, PsycInfo and Medline. We used a broad definition of suicidal ideation and behavior outlined by Posner et al. (2007) and Turecki and Brent (2016). The following keywords were used when extracting articles: (social anx* or social phob* or SAD or social anxiety disorder) and (suicid* or self-injury or self-harm or self-mutil* or self-cut* or cutting or self-burn* or self-poison* or deliberate self-harm or DSH or parasuicid*) and (follow-up or follow up or longitud* or prospective or future or subsequent or epidemio*) and (child* or youth or adol* or young or teen*). We included the term parasuicide (which refers to self-harm behavior without suicidal intent (Welch, 2001)) to reduce the risk of missing relevant studies (in line with the method of Bentley et al (2016)), but studies only examining self-harm behaviour without suicidal intent were not included in the review. Reference lists of included studies were screened to identify relevant articles. Duplicates were removed. A full search electronic search strategy is provided for the Embase in the Supplementary Materials (Appendix 2).

### Study Selection

We followed the Meta-analysis of Observational Studies in Epidemiology (MOOSE) guidelines for the meta-analysis of observational studies. Studies were included if they (1) involved participants who were aged 10–25 years at the first assessment time point, (2) applied at least one measure for social anxiety diagnosis or symptoms and for a dimension of suicidality (e.g., suicidal thought, suicidal ideation, suicidal plan, suicidal behavior, suicidal attempt, suicidal self-injury, death by suicide), (3) reported an outcome measure of the association between social anxiety diagnosis or symptoms and suicidality or examined factors that may underlie suicidality in people with social anxiety diagnosis or symptoms, and (4) were published in English language, peer-reviewed, and indexed scientific journals. Studies were excluded if (5) the study was a review article, a conference abstract or paper, or a research dissertation. Two researchers (EL and KC) independently examined all titles and abstracts to determine whether papers met inclusion criteria. Disagreement was resolved by consensus. Data was extracted independently using an electronic spreadsheet.

### Quality Assessment

Two researchers (EL and KC) assessed the risk of bias independently. The Effective Public Health Practice Project (EPHPP) Quality Assessment Tool for Quantitative Studies (Effective Public Health Practice Project, 2007) was used to assess study quality, as recommended by the Cochrane Collaboration. The EPHPP tool consists of six criteria: selection bias, study design, confounders, blinding, data collection methods, withdrawals and drop-out. Each criterion was assessed on a three-point scale (1 = Strong, 2 = Moderate, 3 = Weak). Each study received an overall rating (Strong = no weak ratings, Moderate = one weak rating, Weak = two or more weak ratings). Discrepancies between assessor-report quality scores were resolved through discussion. The reasons for discrepancy, including oversight, differences in interpretation of criteria, or differences interpretation of study, were recorded. The two assessors rated seven papers together to establish consistency.

### Data Extraction

The following information was extracted from each study and recorded on a data extraction form: research design, source of data, recruitment method, length of follow-up assessment, country, sample size, age, gender, measure used to assess social anxiety, measure used to assess suicidality, type of suicidality outcome, matching and confounding factors.

### Data Analysis

Pearson’s correlation coefficient (*r*) was chosen as the effect size because it is commonly reported in observational studies. For studies that did not report *r,* standardized regression coefficients were converted to *r* (Peterson & Brown, 2005). Odds ratios were transformed to *r* (Borenstein et al., 2010). When studies reported effect sizes for girls and boys separately, effect sizes were combined. All the effect sizes reported were unadjusted.

Meta-analyses were conducted using RStudio (R Core Team, 2019) and the *metafor* package in R (Viechtbauer, 2010). A random-effects meta-analysis model was conducted to examine the association between social anxiety and aspects of suicidality because variations in outcomes between studies were expected due to differences in study characteristics.

Effect size of each study was converted to Fisher’s *Z* for meta-analysis, and the summary Fisher’s *Z* score was converted to a summary correlation. Cohen’s guidelines (Cohen, 1988) were used to interpret the magnitude of effect sizes (*r* = 0.10 ‘*small effect*’, *r* = 0.30 ‘*moderate effect*’, *r* = 0.50 ‘*large effect*’). The Cochran’s *Q* test and the Higgin’s and Thompson’s *I*^*2*^ test were used to assess the degree of heterogeneity between studies. A statistically significant result from the Cochran’s *Q* test (*p* < 0.05) suggests the presence of heterogeneity. A higher *I*^*2*^ value indicates a higher degree of heterogeneity (25% = ‘*low heterogeneity*’, 50% = ‘*moderate heterogeneity*’, 75% = ‘*substantial heterogeneity*’, Higgins et al., 2003).

The risk of bias across studies was evaluated by inspecting the funnel plot and running the Egger’s test (Egger et al., 1997). A significant Egger’s test statistic (*p* < 0.05) suggests there is substantial asymmetry in the funnel plot, and such asymmetry may be indicative of publication bias. Moderator analyses were conducted to ascertain if sample characteristics impacted the effect size estimate. A series of meta-regressions was planned to examine several study characteristics as potential moderators when there were sufficient studies (*k* ≥ 5) in each subgroup: (1) age, (2) gender (coded as the percentage of female participants), (3) publication year, and (4) depressive symptoms.

## Results

### Search Results

**Fig. 1:**
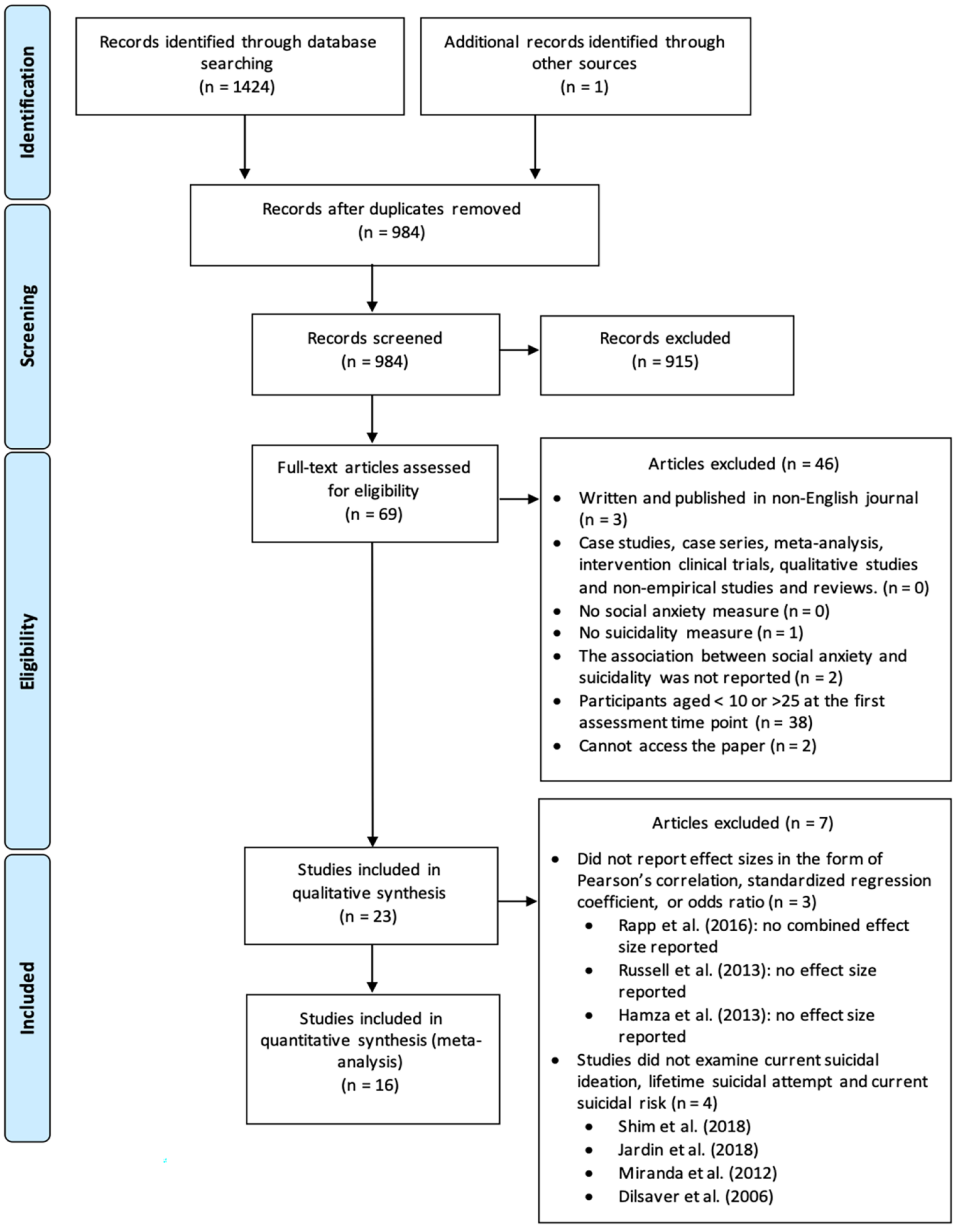
PRISMA diagram

Figure 1 displays the literature search process using a PRISMA diagram (Moher et al., 2009). KC performed the initial literature search and screening. Two coders (KC and EL) reviewed 69 articles independently and the inter-rater reliability was excellent (Kappa coefficient = 0.83). After excluding 46 ineligible studies, 23 studies were retained for quality assessment. Three articles were excluded as the data could not be converted to effect sizes suitable for meta-analysis. Four studies were excluded as they did not measure lifetime suicidal attempt, current suicidal ideation, or current suicidal risk. One additional study was identified when examining the literature (this is included in the total of 69 articles reported above). Therefore, 16 studies were included in the meta-analyses.

### Study Characteristics

Tables 1, 2, 3, 4, and 5 summarise the study characteristics. Sample sizes ranged from 110 to 6483 (*M* = 1729.13, *SD* = 1784.62). Participants were between 12 and 24 years (*M* = 16.59, *SD* = 2.77). Percentages of female participants ranged from 17 to 100% (*M* = 62.53, *SD* = 24.16). Participants were recruited from a university (*n* = 3), a prison (*n* = 1), a hospital (*n* = 2), or the community via flyers, posters, or social media (*n* = 10). Three out of sixteen studies recruited clinical participants (Gallagher et al., 2014; Herres et al., 2019; Strauss et al., 2000). Seven studies were rated as ‘*weak*’ in quality, eight as ‘*moderate*’, and one study as ‘*strong*’.

**Table 1:** Summary of studies examining the cross-sectional association between social anxiety (SA) and lifetime suicidal attempt (LSA)

**Study**	**Sample size**	**Age (range/ mean)**	**Percentage of female participants**	**Country**	**SA measure**	**Variable type**	**Rater**	**LSA measure**	**Variable type**	**Rater**	**Effect size: SA to LSA**	**Quality**
Strauss et al. (2000)	1979	15–19	57.9	US	K-SAD-P	Dichotomous	Clinician	K-SAD-P	Dichotomous	Clinician	0.00	Weak
Nelson et al. (2000)	1344	18.2	100	US	DICA	Dichotomous	Trained interviewer	SSAGA	Dichotomous	Trained interviewer	0.06	Weak
Glowinski et al. (2001)	3416	15.5	100	US	C-SSAGA	Dichotomous	Trained interviewer	C-SSAGA	Dichotomous	Trained interviewer	0.16	Moderate
Nock et al. (2013)	6483	10–17	Not reported	US	CIDI	Dichotomous	Trained interviewer	CIDI	Dichotomous	Trained interviewer	0.18	Moderate
Chabrol et al. (2014)	972	16.9	39	France	SSA	Dichotomous	Self-report	RSIS	Dichotomous	Self-report	0.13	Moderate
Vivar et al. (2014)	991	12–17	51.5	Peru	MINI	Dichotomous	Clinician	MHQ	Dichotomous	Self-report	0.20	Weak
Jahn et al. (2016)	590	18.8	60.5	US	SPAI-23	Continuous	Self-report	BSS	Dichotomous	Self-report	0.01	Moderate
Herres et al. (2019)	115	14.96	83.5	US	C-DISC	Dichotomous	Trained interviewer	C-SSRS	Dichotomous	Trained interviewer	0.03	Moderate

*BSS* Beck Scale for Suicidal Ideation (Beck & Steer, 1991), *C-DISC* Computerized-Diagnostic Interview Schedule for Children (Shaffer et al., 1993), *CIDI* Composite International Diagnostic Interview (Kessler & Ustün, 2004), *C-SSAGA* Child Semi-Structured Assessment for the Genetics of Alcoholism (Reich, 1996), *C-SSRS* Columbia-Suicide Severity Rating Scale (Posner et al., 2011), *DICA* Diagnostic Interview for Children and Adolescents (Reich, 1996), *K-SADS-P* The Schedule for Affective Disorders and Schizophrenia for School Aged Children – Present Episode (Chambers et al., 1985), *MHQ* Mental Health Questionnaire (Perales et al., 1995), *MINI* Mini-international Neuropsychiatric Interview (Sheehan et al., 1998), *RSIS* Revised Suicide Ideation Scale (Rudd, 1989), *SPAI-23* Social Phobia and Anxiety Inventory-23 (Roberson-Nay et al., 2007), *SSAGA* Semi-Structured Assessment for the Genetics of Alcoholism (Bucholz et al., 1994), *SSA* State Social Anxiety Questionnaire (Kashdan & Steger, 2006)

**Table 2:** Summary of studies examining the cross-sectional association between social anxiety (SA) and current suicidal ideation (CSI)

**Study**	**Sample size**	**Age (range/ mean)**	**Percentage of female participants**	**Country**	**SA measure**	**Variable type**	**Rater**	**CSI measure**	**Variable type**	**Rater**	**Effect size:** **SA to CSI**	**Quality**
Strauss et al. (2000)	1979	15–19	57.9	US	K-SAD-P	Dichotomous	Clinician	K-SAD-P	Dichotomous	Clinician	0.04	Weak
Burke et al. (2017)	110	20.13	85.5	US	SIAS	Continuous	Self-report	BSS	Dichotomous	Self-report	0.11	Weak
Heo et al. (2018)	1884	13–16	33.5	Republic of Korea	K-SAS-CA	Continuous	Self-report	RSIQ	Continuous	Self-report	0.47	Weak
Herres et al. (2019)	115	14.96	83.5	US	C-DISC	Dichotomous	Trained interviewer	SIQ-JR	Continuous	Trained interviewer	0.21	Moderate

*BSS* Beck Scale for Suicidal Ideation (Beck & Steer, 1991), *C-DISC* Computerized-Diagnostic Interview Schedule for Children (Shaffer et al., 2000), *IDAS* The Inventory of Depression and Anxiety Symptoms (Watson et al., 2007), *K-SAS-CA* Korean Social Anxiety Scale for Children and Adolescents (Moon & Oh, 2002), *K-SAD-P* The Schedule for Affective Disorders and Schizophrenia for School Aged Children – Present Episode (Chambers et al., 1985), *RSIQ* Reynolds Suicidal Ideation Questionnaire (Reynolds, 1991), *SIAS* Social Interaction Anxiety Scale (Mattick & Clarke, 1998), *SIQ-JR* Suicidal Ideation Questionnaire-Junior (Reynolds & Mazza, 1999)

**Table 3:** Summary of studies examining the cross-sectional association between social anxiety (SA) and current suicidal risk (CSR)

**Study**	**Sample size**	**Age (range/ mean)**	**Percentage of female participants**	**Country**	**SA measure**	**Variable type**	**Rater**	**CSR measure**	**Variable type**	**Rater**	**Effect size:** **SA to CSR**	**Quality**
Plattner et al. (2007)	319	14–21	16.6	Austria	MINI	Dichotomous	Trained interviewer	MINI	Dichotomous	Trained interviewer	0.12	Weak
Jardin et al. (2018)	788	20.83	80.8	US	IDAS	Continuous	Self-report	IDAS	Continuous	Self-report	0.41	Moderate
Gomes et al. (2019)	3781	22	53.4	Brazil	MINI	Dichotomous	Trained interviewer	MINI	Dichotomous	Trained interviewer	0.16	Weak

The IDAS has six items that measure suicidal thoughts and behaviors experienced during the previous two weeks; the MINI contains six questions on suicide risk (wish to be dead, wish to self-harm, suicidal thoughts, suicidal planning, suicide attempt in the past month); a ‘*yes*’ response to any one of these six questions indicates the presence of current suicidal risk

*IDAS* The Inventory of Depression and Anxiety Symptoms (Watson et al., 2007), *MINI* Mini-international Neuropsychiatric Interview (Sheehan et al., 1998)

**Table 4:** Summary of studies examining the prospective association between social anxiety (SA) and lifetime suicidal attempt (LSA)

**Study**	**Sample size**	**Age (range/ mean)**	**Percentage of female participants**	**Time interval (months)**	**Country**	**SA measure**	**Variable type**	**Rater**	**LSA measure**	**Variable type**	**Rater**	**Effect size:** **SA to LSA**	**Quality**
Stein et al. (2001)	3021	14–24	50.59	34–50	Germany	M-CIDI	Dichotomous	Trained Interviewer	M-CIDI	Dichotomous	Trained Interviewer	0.17	Moderate

*M-CIDI* Munich-Composite International Diagnostic Interview (Wittchen et al., 1999)

**Table 5:** Summary of studies examining the prospective association between social anxiety (SA) and current suicidal ideation (CSI)

**Study**	**Sample size**	**Age (range/ mean)**	**Percentage of female participants**	**Time interval (months)**	**Country**	**SA measure**	**Variable type**	**Rater**	**CSI** **measure**	**Variable type**	**Rater**	**Effect size: SA to CSI**	**Quality**
Gallagher et al. (2014)	144	12–15	72	18	US	SAS-A	Continuous	Self-report	SIQ	Continuous	Self-report	0.32	Strong
Zhu et al. (2021)	1491	13.04	53	9	China	LSAS	Continuous	Self-report	PHQ9	Dichotomous	Self-report	0.80	Moderate

*LSAS* Liebowitz Social Anxiety Scale (Heimberg et al., 1999), *PHQ-9* Patient Health Questionnaire-9 (Kroenke et al., 2001), *SAS-A* Social Anxiety Scale for Adolescents (La Greca & Lopez, 1998), *SIQ* Suicidal Ideation Questionnaire (Reynolds, 1985)

Eight cross-sectional studies measured the presence or absence of a lifetime suicidal attempt (Chabrol et al., 2014; Glowinski et al., 2001; Herres et al., 2019; Jahn et al., 2016; Nelson et al., 2000; Nock et al., 2013; Strauss et al., 2000; Vivar et al., 2014). Three studies assessed current suicidal risk, based on questionnaire items capturing both suicidal ideation and attempts (Gomes et al., 2019; Jardin et al., 2018; Plattner et al., 2007). Three prospective studies (Gallagher et al., 2014; Stein et al., 2001; Zhu et al., 2021) examined the relationship between social anxiety and suicidality, with one on suicidal attempt (Stein et al., 2001) and two on suicidal ideation (Gallagher et al., 2014; Zhu et al., 2021). Six studies reported zero-ordered correlations (Burke et al., 2017; Gallagher et al., 2014; Heo, et al., 2018; Herres et al., 2019; Jahn et al., 2016; Jardin et al., 2018). Seven studies reported odds ratios (Glowinski et al., 2001; Gomes et al., 2019; Nelson et al., 2000; Plattner et al., 2007; Stein et al., 2001; Strauss et al., 2000; Vivar et al., 2014), and three studies reported standardized regression coefficients (Chabrol et al., 2014; Nock et al., 2013; Zhu et al., 2021).

### Meta-analysis of T1 Social Anxiety and T1 Lifetime Suicidal Attempt

**Fig. 2:**
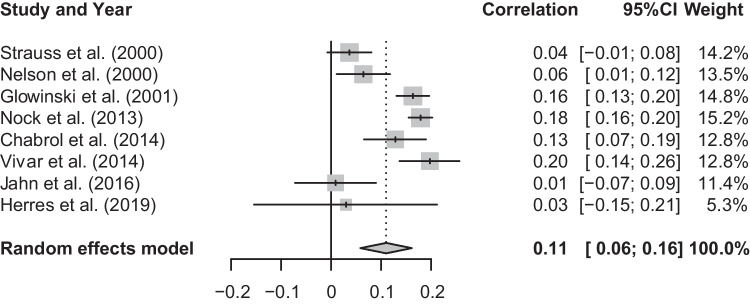
Forest plot of correlations between T1 social anxiety and T1 lifetime suicidal attempt and 95% confidence interval for random effects model

The mean effect size for the meta-analysis examining the association between T1 social anxiety and T1 lifetime suicidal attempt was statistically significant, *r* = 0.10, *p* < 0.05, CI [0.04, 0.15], suggesting that higher levels of social anxiety were associated with more likely presence of suicidal attempt (See Fig. 2). There was a significant and substantial degree of heterogeneity, *Q*(6) = 38.74, *p* < 0.001, *I*^2^ = 84.5%. Neither gender nor publication year were found to be statistically significant moderators: gender (*Q*(1) = 0.01, *p* = 0.92), publication year (*Q*(1) = 0.01, *p* = 0.94). The moderator effects of depressive symptoms and age were not examined due to lack of studies: two studies reported the level of depressive symptoms in their study sample (Chabrol et al., 2014; Jahn et al., 2016) and there were less than five studies to examine age as a moderator.

### Meta-analysis of T1 Social Anxiety and T1 Current Suicidal Ideation

**Fig. 3:**
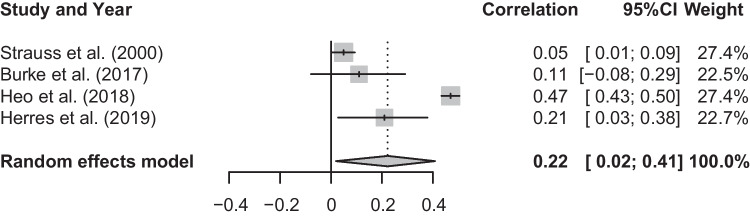
Forest plot of correlations between T1 social anxiety and T1 suicidal ideation and 95% confidence interval for random effects model

The mean effect size for the meta-analysis examining the association between T1 social anxiety and T1 current suicidal ideation was statistically significant, *r* = 0.22, *p* < 0.05, CI [0.02, 0.41], suggesting that higher levels of social anxiety are associated with more frequent suicidal ideation (See Fig. 3). There was a significant and substantial degree of heterogeneity, *Q*(3) = 207.74, *p* < 0.001, *I*^2^ = 98.6%.

### Meta-analysis of T1 Social Anxiety and T1 Current Suicidal Risk

**Fig. 4:**
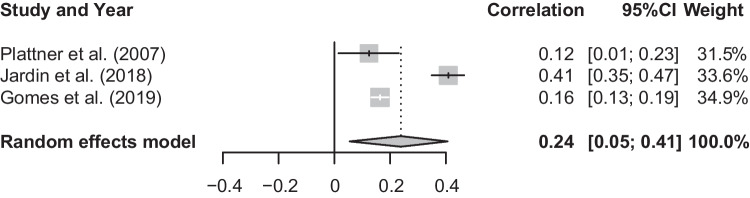
Forest plot of correlations between T1 social anxiety and T1 current suicidal risk and 95% confidence interval for random effects model

The mean effect size for the meta-analysis examining the association between T1 social anxiety and T1 current suicidal risk was statistically significant, *r* = 0.24, *p* < 0.05, CI [0.05, 0.41], suggesting that higher social anxiety is associated with higher current suicidal risk (See Fig. 4). There was a significant and substantial degree of heterogeneity, *Q*(2) = 49.35, *p* < 0.001, *I*^2^ = 95.9%.

### Meta-analysis of T1 Social Anxiety and T2 Suicidal Attempt

Meta-analysis of T1 social anxiety with suicidal attempt in the follow-up period (T1 to T2) was not conducted as only one study examined this association.

### Meta-analysis of T1 Social Anxiety and T2 Suicidal Ideation

**Fig. 5:**
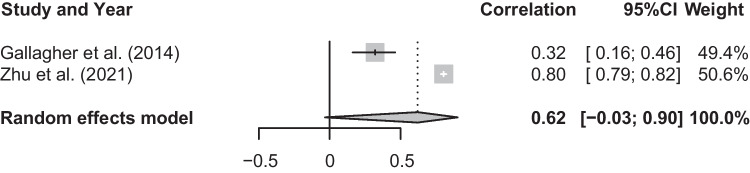
Forest plot of correlations between T1 social anxiety and T2 suicidal ideation and 95% confidence interval for random effects model

The mean effect size for the meta-analysis examining the association between T1 social anxiety and T2 suicidal ideation was not statistically significant, *r* = 0.62, *p* = 0.06, CI [-0.03, 0.90], suggesting that higher levels of social anxiety are not associated with higher T2 suicidal ideation (See Fig. 5). There was a significant and substantial degree of heterogeneity, *Q*(1) = 75.30, *p* < 0.001, *I*^2^ = 98.7%.

### Meta-analysis of T1 Social Anxiety and T2 Current Suicidal Risk

Meta-analysis of T1 social anxiety with T2 current suicidal risk was not conducted due to the absence of relevant studies.

### Publication Bias

Visual inspection of the funnel plot suggests there was a lack of asymmetry in funnel plots for lifetime suicidal attempt (See Appendix 3, Fig. 1). The distribution of the samples for current suicidal ideation, current suicidal risk, and T2 suicidal ideation appeared asymmetrical (See Appendix 3, Figs. 2, 3, and 4). However, none of the Egger’s test statistics was statistically significant (*ps* > 0.05). As such, there is no evidence of publication bias.

### Depressive Symptoms as a Moderator in the Association between Social Anxiety and Suicidality: a Narrative Review of Studies

Four out of sixteen studies examined the relationship between social anxiety and suicidality by controlling for the effect of depressive symptoms or a diagnosis of major depression (Gallagher et al., 2014; Herres et al., 2019; Nelson et al., 2000; Stein et al., 2001). A cross-sectional study conducted by Herres et al. (2019) examined the difference in severity of suicidal ideation between adolescents with major depression only and those with major depression and SAD. They found those with SAD reported significantly more severe suicidal ideation than those without even after controlling for major depression (*F* = 3.99, *p* < 0.05). Another cross-sectional study reported a three-fold increase in risk of suicidal attempt in individuals with major depression who had a history of SAD (Nelson et al., 2000). A prospective study compared the presence of suicidal attempt between those with major depression and SAD and those with major depression only (Stein et al., 2001) and the former group was found to be associated with significantly more suicidal attempts than the latter group (*OR* = 6.1, *p* < 0.05). Using structural equation modelling, Gallagher et al. (2014) found that baseline social anxiety symptoms prospectively predicted suicidal ideation at 18-month follow-up even after controlling for baseline depressive symptoms.

## Discussion

Our review aimed to synthesize findings on the association between social anxiety and suicidal thoughts and behaviors in adolescents (aged 10–25 years). Meta-analyses of 16 studies showed that social anxiety was associated cross-sectionally with suicidality, as measured by suicide attempt, suicidal ideation, and composite suicide risk, and prospectively at trend level with suicidal ideation. The examination of moderator effects was limited by a lack of studies. Only the effect of gender and publication year could be examined, with neither statistically significant, suggesting these factors cannot explain the heterogeneity between studies. Unfortunately the potential moderating effect of age could not be examined due to a lack of studies. It will be valuable to examine this in future studies, given the important potential developmental differences in the association between social anxiety and suicidality. There were several studies suggesting that results could not be purely explained by depressive symptoms but we were unable to test this quantitatively due to the limited number of available studies. Findings therefore provide evidence of a link between social anxiety and suicidal thoughts and behaviors in youth, but most of the studies are cross-sectional and only a few were available for each suicidality construct. This prevented us from drawing conclusions about causality, examining the unique contribution of depression, and explaining the stark heterogeneity among studies.

The quality of the papers included was, on the whole, either weak or moderate, with only one study rated to be of good quality. The quality is partly compromised by the lack of statistical control for covariates, including other anxiety disorders. Thus, it remains unclear whether social anxiety specifically, or anxiety more generally, may increase the risk of suicide in this population. For example, it may be that individuals consider suicide because they feel unable to cope with the worry and anxiety they experience (Sareen et al., 2005). The limitation we confronted in this meta-analysis highlights the need for further well-controlled prospective studies examining the association between these constructs in youth.

As with all suicide research, there was heterogeneity of outcomes measures, ranging from suicidal thoughts to behaviors or more generally “suicide risk”, and it will be helpful for future studies to examine this further. For example, as there are important differences between suicidal ideators and attempters (Klonsky & May, 2014), it will be important to know if social anxiety is particularly related to either or both experiences. Notwithstanding this shortcoming, the consistency of the findings across various measurements of suicidality is encouraging. For all three indices of suicidality, the cross-sectional association was observed to be statistically significant but small in magnitude (*r* < 0.3). Meta-analyses of 50 years of suicide research have indicated that the suicide risk factors evaluated in the literature has stayed somewhat consistent (with ‘internalizing disorders’ as the most commonly studied), with limited improvement in the ability to predict suicidal behavior over decades (Franklin et al., 2017). Therefore, we focused on social anxiety as a potential modifiable risk factor because we have cognitive conceptualizations and treatments for social anxiety which could potentially better identify as well as reduce suicide risk in youth (Chiu et al., 2021a; Leigh & Clark, 2018, 2022).

Whereas suicidal behavior has been linked to a wide range of psychiatric diagnoses, social anxiety may be particularly relevant to understanding the etiology of suicidal thoughts and behaviors. Psychological theories of suicide often overlap with core constructs in social anxiety, such as beliefs about unacceptability and social disconnection (Clark & Wells, 1995; Leigh & Clark, 2021). For example, the interpersonal theory of suicide (Joiner et al., 2005) suggests suicidal desire emerges from thwarted belongingness and perceived burdensomeness. Thwarted belongingness refers to the distressing state that results when the “need to belong” and the desire for social connection are not met. Perceived burdensomeness reflects a sense that others would be “better off if I were gone.” These psychological constructs will be influenced by both environmental and psychological factors. These include the individual’s actual interpersonal environment, for example, social isolation due to peer victimisation or a lack of reciprocal care arising from family conflict, and also their beliefs about their interpersonal world, for example, perceptions of peer rejection. Socially anxious individuals tend to hold excessively negative views about their social acceptability and in this way may be more vulnerable to experiencing a sense of social disconnection and perceived burdensomeness. Likewise, in O’Connor’s integrated-volitional model of suicide, defeat and humiliation are key drivers in the motivational phase of suicidal ideation and intention formation (O'Connor & Nock, 2014) and the hallmark feature of social anxiety is fear of embarrassment and humiliation. Relatedly, while there are a wide range of risk factors and warning signs for suicidal behavior, the six hours before a suicide attempt is often characterized by negative interpersonal event, along with the affective responses such as burdensomeness, feeling scared and empty (Bagge et al., 2022). Therefore, the perception of social relationships and social context are key factors in suicide risk, and we know that social anxiety is associated with a tendency to perceive social interactions more negatively (Chen et al., 2020; Chiu et al., 2021b) and experience more shame after social interactions (Schuster et al., 2021). Future studies using multiple measurement points and measurement methods will shed light on the dynamic temporal relationships that likely exists between social anxiety symptoms, suicidal thoughts and behaviours, and inter- and intra-personal factors.

Our study built on the meta-analysis by Bentley et al. (2016) by including eight additional studies. Strengths of the present study include the rigor of our methods, including both cross-sectional and longitudinal analysis as well as multiple outcomes related to suicidal thoughts and behaviors. There are limitations to consider. We were unable to undertake some of the planned moderator analyses due to insufficient studies and so we do not know the source(s) of the observed heterogeneity. We only included English language studies due to limited resources, although it is unlikely that this affected our findings.

The present findings suggest that we may be able to reduce suicidal thoughts and behaviors in adolescents via early identification and treatment of social anxiety. Due to the low rates of treatment-seeking in SAD (Olfson et al., 2000) active screening programmes, perhaps in schools may be needed to identify young people struggling with social anxiety symptoms. In terms of interventions, encouragingly we do have effective treatments for SAD. For example, Cognitive Therapy for SAD, is recommended as a first-line treatment for adult SAD by UK clinical guidelines (National Institute for Health and Care Excellence, 2013) and the treatment is associated with large, controlled effect sizes with adolescents (Ingul et al., 2014; Leigh & Clark, 2022). However, because only three studies included in the review recruited from clinical populations caution should be taken when generalizing the findings to adolescents with a clinical diagnosis of SAD.

The current review highlights the ongoing need for well-controlled prospective studies of social anxiety symptoms/disorder and suicidal thoughts and behaviors in youth. These will allow us to understand the specificity and temporality of the association between the two constructs. If confirmed, subsequent studies could address the underlying mechanisms, which may include psychological factors such as loneliness (Gallagher et al., 2014) and negative self-perceptions (Clark & Wells, 1995), environmental factors such as interpersonal stress, and their interaction (Joiner et al., 2005). A further avenue for future research includes examination of the association between social anxiety/SAD with suicidal thoughts and behaviors across different populations, including pre-adolescents, genders, and ethnicities. Understanding the association amongst those who are at greater suicide risk, such as LGBTQ+ populations, will also be important clinically.

The present review provides evidence for the contributory role of social anxiety to suicidal thoughts and behaviours in youth aged 10 to 25 years but also highlights the need for further high quality prospective studies. As stated previously, with the global burden of suicide, it is critical to examine modifiable risk factors for suicide including social anxiety that can inform future treatments for suicidal youth.

## Supplementary Information

Below is the link to the electronic supplementary material.

Supplementary file1 (DOCX 155 KB)
